# Multiple extracellular polymeric substance pathways transcribed by *Accumulibacter* and the flanking community during aerobic granule formation and after influent modification

**DOI:** 10.1128/aem.01769-24

**Published:** 2025-03-31

**Authors:** Laëtitia Cardona, Jaspreet Singh Saini, Pilar Natalia Rodilla Ramírez, Aline Adler, Christof Holliger

**Affiliations:** 1 Laboratory for Environmental Biotechnology, Ecole Polytechnique Fédérale de Lausanne 27218, Lausanne, Switzerland; Washington University in St. Louis, St. Louis, Missouri, USA

**Keywords:** metatranscriptomics, sequencing batch reactor, *Propionivibrio*, 16S rRNA gene amplicon sequencing, enhanced biological phosphate removal, phosphate-accumulating organisms

## Abstract

Aerobic granular sludge is a biological wastewater treatment process in which a microbial community forms a granular biofilm. The role of *Candidatus* Accumulibacter in the production of a biofilm matrix composed of extracellular polymeric substances was studied in a sequencing batch reactor enriched with polyphosphate-accumulating organisms. The metabolisms of the microbial populations were investigated using *de novo* metatranscriptomics analysis. Finally, the effect of decreasing the influent phosphate concentration on the granule stability and microbial activity was investigated. A few weeks after the reactor start-up, the microbial community was dominated by *Accumulibacter* with up to nine species active in parallel. However, the most active species differed according to sampling time. Decreasing drastically the influent phosphate concentration led to a dominance of the glycogen-accumulating organism *Propionivibrio*, with some *Accumulibacter* species still abundant. *De novo* metatranscriptomics analysis indicated a high diversity of potential extracellular substances produced mainly by *Accumulibacter*, *Azonexus*, *Candidatus* Contendobacter, and *Propionivibrio*. Moreover, the results suggest that *Azonexus*, *Contendobacter,* and *Propionivibrio* recycle the neuraminic acids produced by *Accumulibacter*. Changes in the microbial community did not cause the granules to disintegrate, indicating that a *Propionivibrio*-dominated community can maintain stable granules.

**IMPORTANCE:**

One of the main advantages of the aerobic granular sludge wastewater treatment process is the higher settling velocities compared to the conventional activated sludge-based process. In aerobic granular sludge, the biomass is concentrated into a biofilm matrix composed of biopolymers, providing micro-niches to different types of microbial populations. We demonstrate with the help of *de novo* metatranscriptomics analysis that the formation of granules is a highly dynamic microbial process, even when enriching for a microbial guild, such as phosphate-accumulating organisms. Often underestimated, the flanking community of the main phosphate-accumulating organisms population enriched in the reactor is nonetheless active and transcribing genes related to different extracellular polymeric substance pathways. The multiplicity of the extracellular polymeric substances produced probably helped the matrix to remain stable, thanks to their specific properties. Moreover, the results suggest microbial interactions in extracellular polymeric substance recycling between different microbial populations that can be helpful to prevent a disruption of the granules while stressing out the microbial community.

## INTRODUCTION

The removal of phosphorus from wastewater is essential to avoid eutrophication of the natural environment and can be part of the circular economy by recovering phosphorus to be used as a fertilizer. The enhanced biological phosphorus removal (EBPR) process, operated in sequencing batch reactors (SBRs), allows the removal of phosphorus from wastewater. In EBPR-SBRs, anaerobic feeding and aerobic starvation phases are alternated, which favors the enrichment of phosphate-accumulating organisms (PAO) responsible for EBPR. These operation conditions can also lead to the enrichment of glycogen-accumulating organisms (GAO) that compete with PAO for the carbon source. During the anaerobic feeding phase, PAO and GAO take up the carbon source and store it intracellularly as polyhydroxyalkanoate (PHA). To provide energy and reduce equivalents for PHA production, PAO consumes intracellular polyphosphate and glycogen, leading to the release of inorganic phosphate into the bulk liquid, whereas GAO only relies on glycogen. During the aerobic starvation phase, PHA is used as a carbon and energy source for growth and to replenish the reserves of polyphosphate (PAO only) and glycogen (both) (1).

EBPR is also used in aerobic granular sludge (AGS) technology in which the microbial community grows in a self-supported granular biofilm. In comparison with the conventional activated sludge technology, AGS allows high biomass concentration, leading to compact reactors and decreasing the operation time, thanks to the fast settleability of the biomass (2). The granular biofilm is composed of a matrix of extracellular polymeric substances (EPS) that are synthesized and excreted by the microbial community. EPS comprise polysaccharides, proteins, nucleic acids, and other molecules (3). Destabilization of the EPS matrix can result in a disintegration of the biofilm and, thus, a decrease in the fast settleability of the biomass, leading to the washout of the microbial community and a reduction in nutrient removal performance. Despite the importance of EPS in the AGS process, there is little information regarding the microbial producers, regulators, and dynamics of the gene transcription, particularly during granulation. Previous efforts to identify the EPS producer relied on isolation (4) and metagenomic studies. In a recent study, Dueholm et al. (5) compared the genetic potential of activated sludge bacteria to produce exopolysaccharides based on their metagenome-resolved genomes (5). They identified specific operons in some of the most common microorganisms also present in AGS, such as the poly-N-acetylglucosamine operon in *Candidatus* Accumulibacter (a PAO, referred to as *Accumulibacter*) and the Pel operon in *Propionivibrio* (a GAO). A few studies conducted in SBRs have shown that certain types of EPS are associated with enriched microbial populations. For example, exopolysaccharide ‘granulan’ has been extracted from *Candidatus* Competibacter-enriched biomass (6), while complex glycoconjugates were identified in granules dominated by ammonium-oxidizing bacteria (7). However, these studies mainly focused (i) on the dominant or cultivable microbial population, and, therefore, underestimate the flanking community in the EPS production, (ii) characterized mainly one kind of EPS in each experiment, and (iii) studied the EPS on already formed granules. Hence, only a few studies have focused on EPS producers during AGS formation (8, 9). To fill this knowledge gap, we explored the use of *de novo* metatranscriptomics analysis to identify the active members of the AGS microbial community while forming granules, particularly regarding EPS gene transcription. Additionally, once the granules were formed, we studied the influence of drastically decreasing the phosphate concentration on granule stability, microbial community activity, and gene transcription.

## RESULTS

### Nutrient removal performances

**Fig 1:**
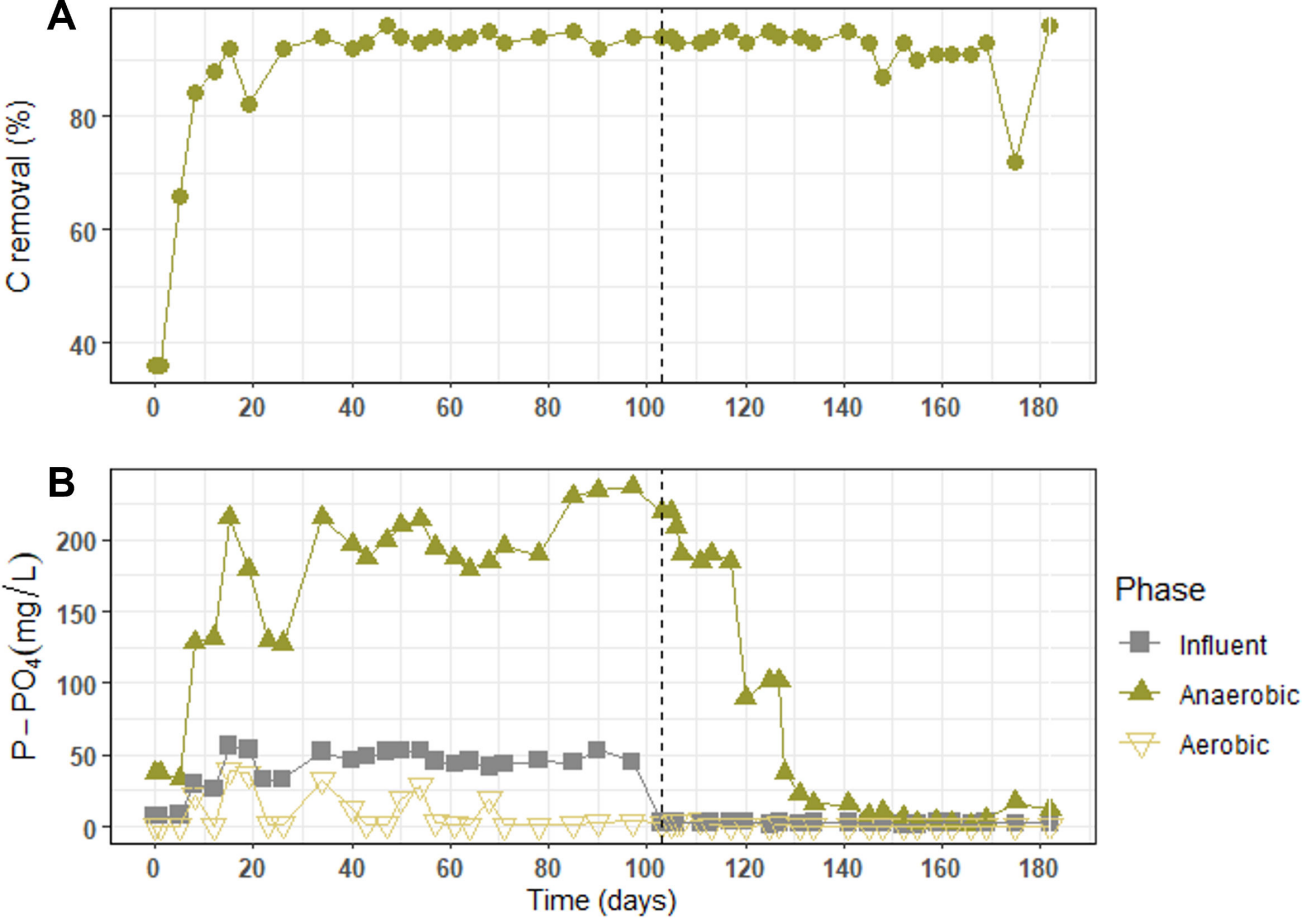
Nutrient removal and efficiencies during the 183 days of reactor operation. (**A**) Carbon removal efficiency during the anaerobic phase. (**B**) Phosphate concentration at the different phases: influent, end of anaerobic, and end of aerobic phase. The dashed line at day 103 indicates the change in the influent composition from COD/P of 12 to 200.

In this study, the carbon removal efficiency was always higher than 90% after an adaptation phase of 15 days (Fig. 1A). As expected for a typical PAO metabolism, the phosphate concentration increased during the anaerobic phase, reaching up to 237 mg/L, and then decreased to as low concentrations of 0.3 to 32 mg/L during the aerobic phase (Fig. 1B). When the influent phosphate concentration was drastically reduced (day 103), the value at the end of the anaerobic phase remained high. This was most probably due to the polyphosphate reserves of PAO. After the conditioning cycles on days 120 and 127, where phosphate-rich bulk liquid was removed at the end of the anaerobic phase, the phosphate release progressively decreased to 1–15 mg/L at the end of the anaerobic phase and remained low for the last 5 to 6 weeks of reactor operation.

### Granulation and microbial community composition dynamics

**Fig 2:**
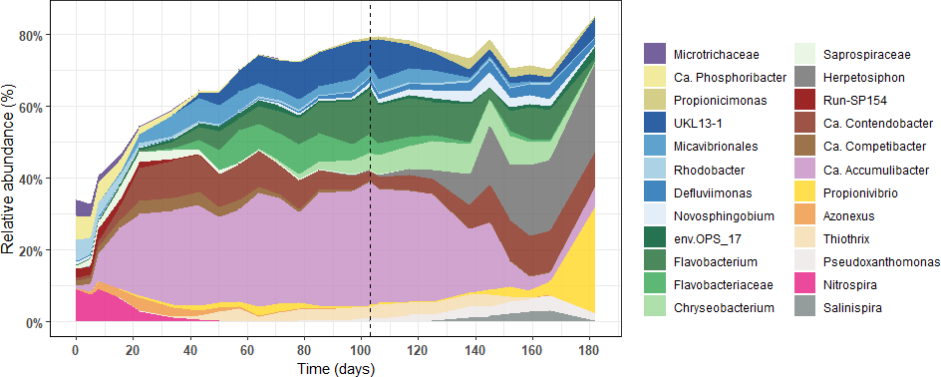
Relative abundance of the most abundant microorganisms. Relative abundance of the most abundant genera (minimum 3% in at least one sample) based on the 16S rRNA gene amplicon sequencing. The gray dashed line at day 103 indicates the change in the medium composition (from COD/P of 12 to COD/P of 200). Genera are colored based on the class level, with the exception of *Accumulibacter* and *Propionivibrio* highlighted in purple and yellow, respectively.

The relative abundance of different populations of the microbial community is represented in Fig. 2. At the early beginning of the experiment, the microbial community was more diverse and mainly composed of *Candidatus* Phosphoribacter (formerly *Tetrasphaera* clade III [10]), *Nitrospira*, a nitrite-oxidizing organism, the metabolically versatile *Rhodobacter*, and many other microorganisms with a lower abundance. After 1 week of reactor operation, the relative abundance of these microorganisms decreased, while that of the PAO *Accumulibacter* (40%), *Flavobacterium* (10%), the GAO *Candidatus* Contendobacter (10%), Hyphomonadaceae UKL13-1 (10%), and a few other uncharacterized genera from the Flavobacteriaceae and Micavibrionales increased.

After the influent phosphate concentration was lowered at day 103, the microbial community composition slightly changed during the first 20 days. A progressive increase in the relative abundance of *Herpetosiphon* (25%), *Salinispira* (3%), and *Pseudoxanthomonas* (3%) was observed. From day 125 onwards, the relative abundance of *Accumulibacter* progressively decreased to 5%, while that of *Propionivibrio* increased to 28%.

The aspect of the biomass changed across the experiment (Figure A1). From the initial flocs and little aggregates smaller than 200 µm (diameter), the biomass formed tiny smooth granules that grew to over 1,000 µm in diameter. The aspect of the granules changed from smooth to grainy during the low-phosphate period, but the granule stayed intact.

### Active members of the microbial community

In order to identify the active microbial community members at different stages of granulation, metatranscriptomics samples were taken at day 13 (mean particle size around 172 µm, COD/P ratio of 12), day 26 (300 µm, COD/P ratio of 12), day 103 (>1,000 µm, COD/P ratio of 12), and day 182 (>1,000 µm, COD/P ratio of 200).

**Fig 3:**
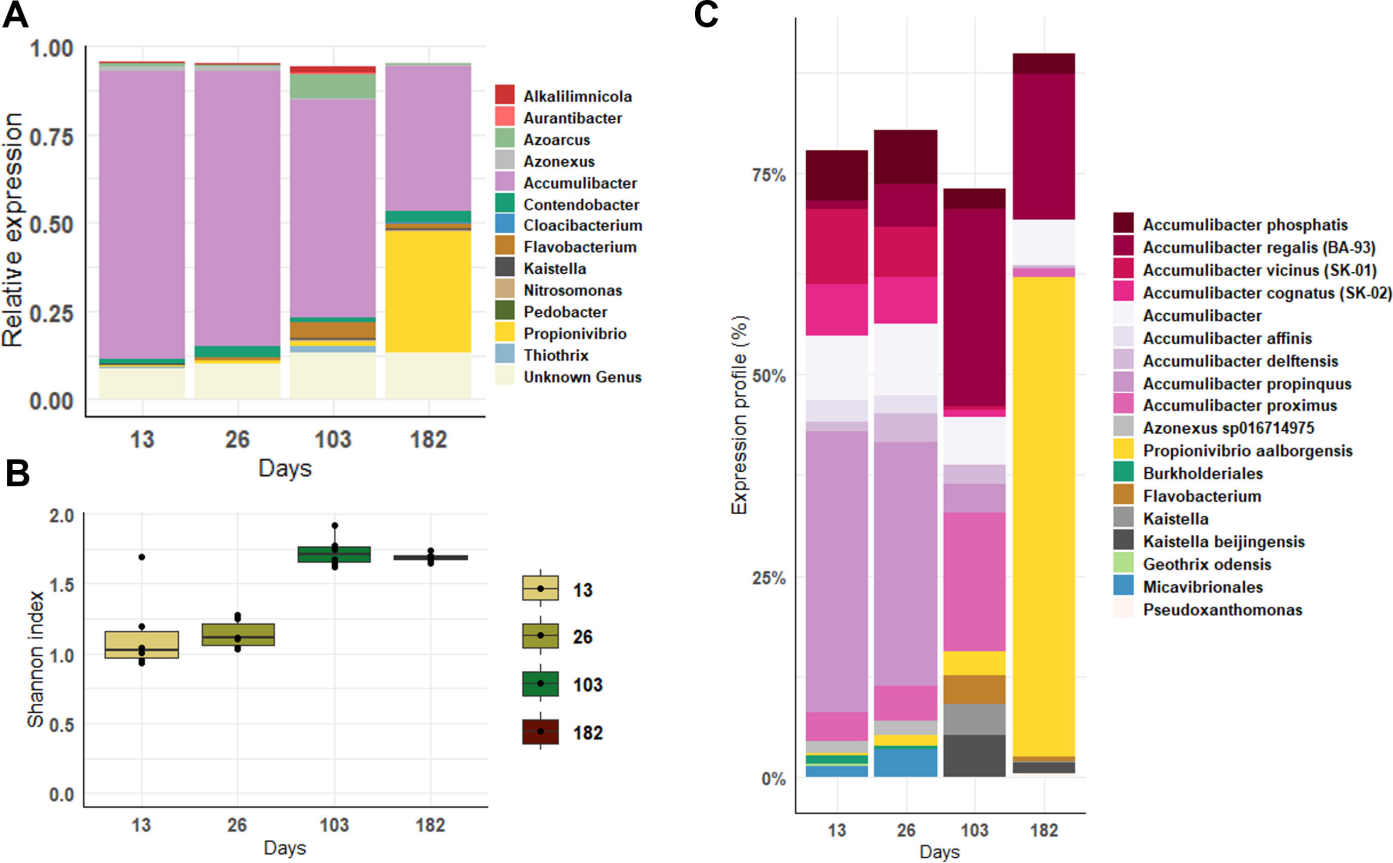
Active microbial population at different stages of granulation and influent composition. (**A**) Relative proportion of the gene expression per genus from the *de novo* metatranscriptomics analysis. Only the 20 most abundant genera are represented. (**B**) Shannon diversity index calculated using the level of expression of each genus. (**C**) Expression profile at the species level of the most abundant microorganisms using mOTUs (minimum of 3% in one sample).

When the granules were still small (days 13 and 26), *Accumulibacter* was the predominant active population accounting for 70% of the transcripts (Fig. 3A). When the granules were larger (day 103), *Accumulibacter* remained predominant, but other active populations, such as *Flavobacterium*, *Kaistella*, *Thiotrix*, and *Azoarcus*, emerged. The Shannon diversity index confirmed the increase of the active members’ diversity with time and the granule size increase (Fig. 3B).

At the end of the low-phosphate period (day 182), *Accumulibacter* and the GAO *Propionivibrio* were the dominant active populations. Although the conditions were favorable for the enrichment of GAO, only *Propionivibrio* activity increased, while the gene transcription of *Contendobacter*, another GAO detected in all the samples, remained low.

The co-occurrence of multiple active species of *Accumulibacter* was observed at the different stages of granulation (Fig. 3C). Interestingly, a shift in the main active ones was observed between days 26 and 103. While *Accumulibacter propinquus* was the most active species in small granules, *Accumulibacter regalis* BA-93 and *Accumulibacter proximus* predominated in large granules. During the low-phosphate period, *A. regalis* BA-93 remained the main active PAO.

### Transcription of metabolic pathways involved in EBPR

**Fig 4:**
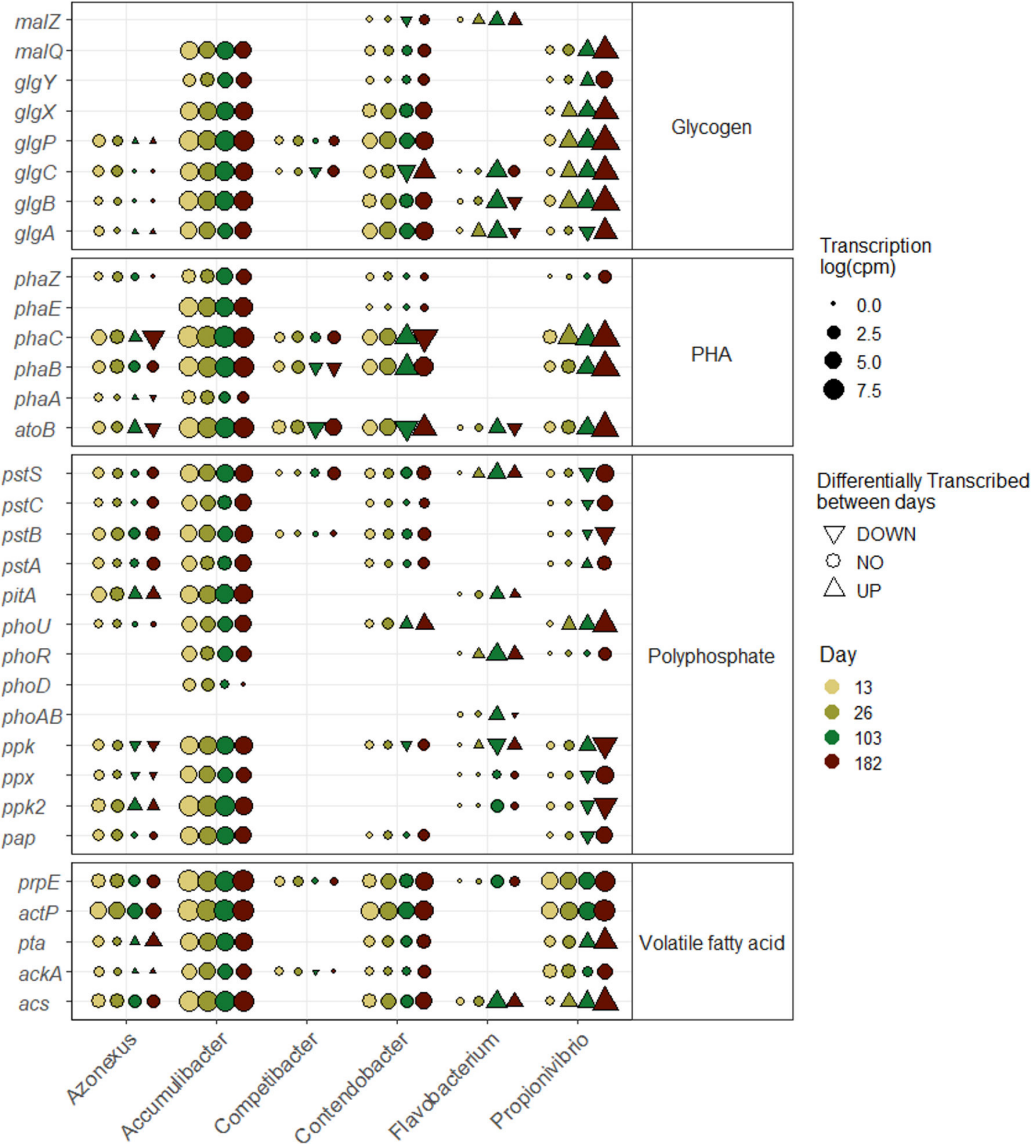
Level of transcription of genes (log(cpm)) of metabolisms involved in the enhanced biological phosphate removal process at the different time points of sampling taken during the feeding phase. Differential gene expression analysis was done between two time points (26 vs 13, 103 vs 26, and 182 vs 103), and the significant differences (log-2 fold change > 2 and adjusted *P* value < 0.01) are represented by a triangle (up-pointing for higher expressed genes compared to the day before and down-pointing triangle for lower expression).

The microorganisms involved in metabolic pathways typical for the EBPR process were identified by analyzing the transcription of the genes related to acetate and propionate uptake and activation and the metabolisms of polyhydroxyalkanoate (PHA), glycogen, and polyphosphate (Fig. 4). *Accumulibacter*, *Contendobacter*, and *Propionivibrio* were the main genera involved in these processes. Transcription of only a few genes of the different metabolisms by *Candidatus* Competibacter (GAO) was detected probably due to its low abundance throughout the experiment.

*Contendobacter* and *Propionivibrio* transcribed polyphosphate-related genes, except the low-affinity transporter *pitA*. While the overall level of transcription of *Contendobacter* did not increase along the experiment, genes related to the EBPR process of *Propionivibrio* had a very high level of transcription at the end of the low-phosphate period.

In this experiment, *Azonexus* transcribed genes related to acetate and propionate uptake (*actP* and *prpE*, respectively) and activation (*acs*, *ackA*, and *pta*), high- and low-phosphate affinity transporters (*pstABCS* and *pitA*, respectively), polyphosphate kinases (*ppk* and *pap*), genes involved in PHA formation (*phaA*, *B* and *C*) and degradation (*phaZ*), and in glycogen branching (*glgABC*) and debranching (*glgP*).

Besides known PAO and GAO genera, *Flavobacterium* also transcribed genes from EBPR pathways (i.e., polyphosphate metabolism, glycogen branching, and propionate transport). The level of transcription for the different genes in *Flavobacterium* increased over time, while the phosphate concentration was high. However, when the influent phosphate was low, the level of transcription decreased.

### Transcription of extracellular polymeric substance genes

**Fig 5:**
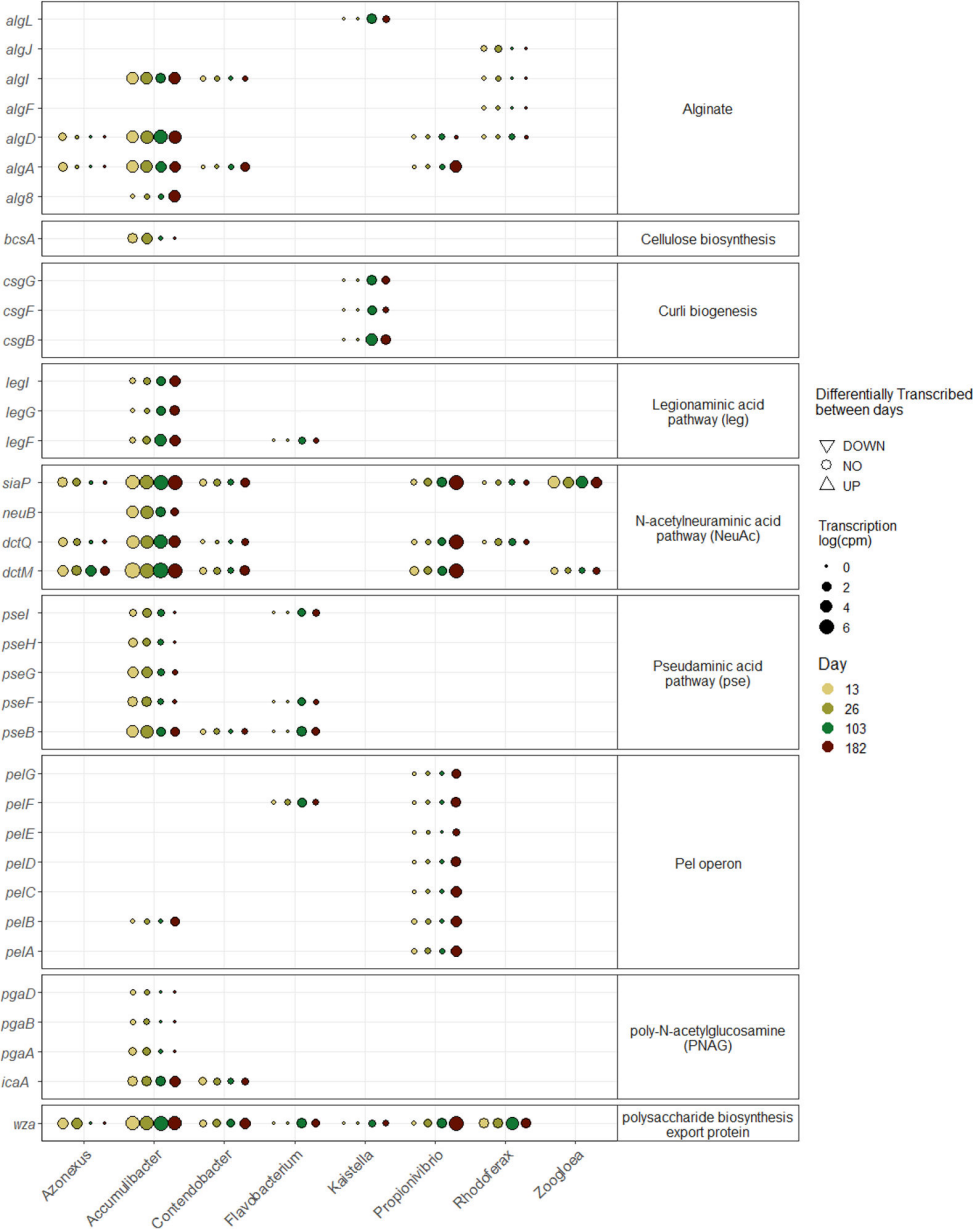
Transcription of genes related to extracellular polymeric substances (EPS) production at different time point sampling. Level of expression (log(cpm)) of genes per day for different genera expressing genes involved in EPS production. Differential gene expression analysis was done between two time points (26 vs 13, 103 vs 26, and 182 vs 103), and the significant differences (log-2 fold change > 2 and adjusted *P* value < 0.01) are represented by a triangle (up-pointing for higher expressed genes compared to the day before and down-pointing triangle for lower expression).

At all stages, *Accumulibacter* transcribed genes from nonulosonic acids [N-acetylneuraminic acid, legionaminic acid (leg), and pseudaminic acid (pse)], cellulose, alginate, and poly-N-acetylglucosamine (PNAG) pathways (Fig. 5). Interestingly, genes for cellulose, PNAG and pse pathways decreased with time and the granule size increase, while the change in the medium composition did not seem to influence their transcription. In contrast, genes of the leg pathway increased. In parallel to transcribing the nonulosonic synthase (*neuB*), *Accumulibacter* also transcribed the neuraminic acid-binding protein (*siaP*), permease (*dctM*), and tripartite ATP-independent periplasmic transporter proteins (*dctQ*) that enable the import of neuraminic acid. Similarly, *Propionivibrio*, *Azonexus*, and *Contendobacter* also transcribed the genes related to the recycling of neuraminic acids but not *neuB*.

Different populations seemed to be involved in the production of other EPS at different stages of the experiment. Most major populations transcribed the polysaccharide biosynthesis export protein *wza*, common to gram-negative bacteria (11). *Propionivibrio* transcribed the pel cluster genes; *Rhodoferax* and *Azonexus* transcribed some genes of the alginate pathway mostly at the beginning of the experiment; and transcription of genes from the curli biogenesis pathway by *Kaistella* was also detected.

Finally, *Zoogloea* transcribed only two genes from the neuraminic acid pathway. However, the overall transcription of *Zoogloea* was lower than 0.01%, and if it played a role during the granulation, it was not captured in the metatranscriptome.

## DISCUSSION

### Microbial population dynamics during aerobic granulation

The goal of this study was to investigate the dynamics and the potential role of a microbial community enriched in classical PAO, as they are at the base of the EBPR process and suggested to be the major player in granule formation. From the beginning of the experiment, starting with a floccular biomass to the formation of the aerobic granules, the microbial community composition highly changed. The microorganisms present in the inoculum, such as *Candidatus* Phosphoribacter or *Nitrospira*, were replaced by the PAO *Accumulibacter* as the major active microorganisms. The use of specific operational conditions (pH 7, 18°C) and medium composition (acetate, propionate, and high phosphate concentration) favored the enrichment of *Accumulibacter* over fermentative PAO, such as *Phosphoribacter* and GAO (12). Concurrently to this enrichment, round granules formed and increased in size. The Shannon index showed an increase in the genus diversity among active members with time and the formation of larger granules. Similarly, Tan et al. (13) noted this phenomenon using complementary DNA while following aerobic granule formation from flocs and suggested that micro-niches within larger granules support a wider variety of bacterial populations (13).

Various *Accumulibacter* species were active at different granulation stages, with species abundance changing over time and as granules grew. This co-occurrence of *Accumulibacter* species has already been noted in full-scale plants (14) using clade-specific qPCR primers and in laboratory SBRs (15) using metagenomic analysis. Both studies observed species dynamics related to temperature and influent composition, respectively. In this study, species-level transcription profiles confirmed the presence and the variation of *Accumulibacter* species over time and granule size increase.

Microorganisms were active alongside *Accumulibacter*, albeit in lower abundance. Among the flanking microbial community, it can be mentioned that the two GAO *Contendobacter* and *Propionivibrio*, which, like PAO, can take up acetate and propionate during the anaerobic feeding phase but only relying on glycogen, were also active. *Azonexus*, previously called *Dechloromonas*, was detected as an active population mainly in the samples of the first two time points. Some *Azonexus* species, such as *A. phosphorivorans* and *A. phosphoritropha*, were defined as PAO based on metagenomic analysis coupled to Raman microspectroscopy quantifying intracellular storage polymers (16). In this study, *Azonexus* transcribed genes for phosphate transport and accumulation, as well as PHA and glycogen metabolisms, indicating that *Azonexus* was involved in the EBPR process.

It is noteworthy that *Flavobacterium* exhibited increased activity during granulation and transcribed genes related to the EBPR metabolism, particularly those involved in phosphate metabolism, volatile fatty acid activation, and glycogen branching. However, no genes from the PHA metabolism were identified as being transcribed. It was suggested that *Flavobacterium* plays a role in phosphate accumulation in the wetland environment from which this bacterium was isolated (17) based on the presence of the genes related to phosphate accumulation *pitA*, *pstSCAB*, *ppk*, and *ppx*. Additionally, the presence of the glycogen-branching genes *glgABC* has been reported in psychrophilic species and proposed as a means to resist low temperatures (18). Despite their prevalence in activated sludge (19), the role of *Flavobacterium* in AGS remains unclear. The transcription of the genes related to phosphate is not an indicator for *Flavobacterium* being a PAO, as phosphate is an important biogenic element for all microorganisms, and the genes related to phosphate accumulation are widespread among the microorganisms. If *pitA*, the gene for a low-affinity phosphate transporter, was mainly identified in PAO, it has also been identified in some GAO, such as *Defluviicoccus* (20), and some genomes of *Propionivibrio* (21). Further investigation is warranted to elucidate the significance of *Flavobacterium* in AGS, such as analyzing the accumulation of the storage polymer using Raman microspectroscopy.

### *Accumulibacter* and *Propionivibrio* as main active populations under low-phosphate conditions

The phosphate concentration is one major parameter for the enrichment of PAO. Slow-growing microorganisms, and more specifically PAO, were suggested to be the main actors for the granule formation and stability (22). Once the nutrient removal performances were stabilized, and large granules were formed, the influent phosphate concentration was decreased (day 103) to either favor the development of GAO or evaluate the capacity of *Accumulibacter* in GAO mode to maintain the biofilm in granular form.

When significantly decreasing the influent phosphate concentration, the anaerobic phase’s phosphate levels remained high, and the microbial community, dominated by *Accumulibacter*, did not change, likely due to PAO’s polyphosphate reserves. The phosphate concentration only decreased after two anaerobic post-drainage cycles to remove the phosphate released. After this phosphate removal measure, there was no more phosphate release/uptake, but carbon removal was still efficient during the anaerobic phase, which indicated a typical GAO metabolism (23). The microbial community composition and activity analyses supported this hypothesis, with the GAO *Propionivibrio* becoming abundant and active. Interestingly, the GAO *Contendobacter* present since the beginning maintained a constant relative abundance and activity, suggesting it was less competitive than *Propionivibrio* under optimal GAO conditions. This may be due to operational conditions, presence of propionate, pH, temperature, or SBR phase operations being sub-optimal for *Contendobacter* development.

Based on the 16S rRNA gene amplicon sequencing results, relative abundance of *Herpetosiphon* increased after the change in the influent composition. *Herpetosiphon* are ubiquitous, filamentous bacteria and have already been identified in PAO-enriched reactor (24). They also have the ability to prey on other microorganisms (25). However, from the metatranscriptomics results, *Herpetosiphon* was barely active, and the role of this genus was then impossible to display. The discrepancy between the two analyses can be explained by the bias of each method (different extractions, library preparation, sequencing depth) but could also be part of the difference between being present and active.

*Accumulibacter* species are divided into two types, called types I and II, based on the polyphosphate kinase gene (ppk1) and supported by genome-resolved phylogenies (21). In the current study, mOTUs profiling results revealed that the *Accumulibacter* population was composed of a mixture of types I (*A. regalis*, *A. delftensis*) and II (*A. propinquus*, *A. proximus*) species being active before the influent phosphate concentration decreases. Under low-phosphate conditions and once the anaerobic phosphate release did not occur anymore, the type I *A. regalis* remained the most active. Indications have been obtained that both *Accumulibacter* types I and II can shift their metabolism from PAO to GAO under different conditions, such as low phosphate concentration (23–25), with type I being less competitive due to a lower acetate uptake rate under polyphosphate-depleted conditions (24). The present results indicate that *Accumulibacter* type I is capable of adapting when the phosphate reserve depletion is not forced.

### Multiple EPS pathways transcribed by *Accumulibacter* and the flanking community

Genes from multiple EPS pathways were transcribed during the granulation process, suggesting a wide variety of EPS during the granule formation. The complexity and the presence of multiple EPS in AGS, as well as changes in the composition, have been previously highlighted (24). The different EPS certainly play different roles in the formation and stability of the biofilm matrix due to their different properties. For example, PNAG is partially N-acetylated polysaccharides that can influence synthesis, translocation, and adhesiveness depending on the microorganisms (3), or De Graff et al. (2019) demonstrated that sialic acids protect the EPS matrix from enzymatic degradation by binding to terminal positions in carbohydrate chains (26). Although the EPS were not analyzed in this present work, metatranscriptomics analysis allowed identification of the potential microbial EPS producers from the flanking community of a PAO-enriched reactor.

*Accumulibacter* transcribed genes from multiple EPS pathways producing PNAG, nonulosonic acids (NulOs), cellulose, or alginate in all time points, and most of them confirmed results previously described. Dueholm et al. (5) identified PNAG gene clusters in *Accumulibacter* genomes, as well as a Pel operon in *Propionivibrio*.

Tomás-Martínez et al. (27) identified genes related to the NulOs pathways from *Accumulibacter* genomes and their expression with proteomics (27). NulOs are nine-carbon monosaccharides primarily found in eukaryotes and pathogenic bacteria. Common types include neuraminic and ketodeoxynonulosonic acids, with legionaminic and pseudaminic acids specific to bacteria. During the granulation process, an inverse transcription trend between pseudaminic and legionaminic acid genes was observed. This could be the result of either a change in the NulOs regulation with time or influenced by the increase of the granule size or by the dynamics of different *Accumulibacter* populations with time. It is possible that not all *Accumulibacter* transcribed the same NulOs genes. Indeed, Tomás-Martínez et al. (27) have shown that most of the *Accumulibacter* metagenome-assembled genomes (MAGs) contain the NeuAc synthase gene but not all either due to incomplete MAGs or to a true genetic absence.

Additionally, the transcription of neuraminic acid binding proteins, permease, and tripartite ATP-independent periplasmic transporter proteins indicates that *Accumulibacter* recycles NulOs to conserve energy, as suggested by the proteomics analysis (27). Similarly, *Propionivibrio*, *Azonexus*, and *Contendobacter* transcribed genes for recycling neuraminic acids but not for NeuAc synthase, suggesting that they reuse NulOs produced by *Accumulibacter*. Further genomic investigation could confirm the presence of the NeuAc gene, indicating potential but unused capability for neuraminic acid production. *Flavobacterium* transcribed some genes of the pseudaminic acid pathway. However, it is not so clear from the literature and the results if they are involved in sialic acid production.

The metatranscriptomics results showed that besides *Accumulibacter*, other microorganisms (*Rhodoferax*, *Propionivibrio*, *Contendobacter*, and *Azonexus*) transcribed genes from other EPS types, such as alginate (ALE), which is an anionic linear polysaccharide composed of mannuronic acid and guluronic acid residues. Mature granules exhibit a higher concentration of ALE in most of the studies and could be the results of the microbial compression during granule maturation (28). A higher ALE content was usually associated with the presence of PAOs and GAOs, such as *Accumulibacter* and *Propionivibrio*, respectively (28). Curli proteins or amyloid adhesins were transcribed by *Kaistella* mostly when the granules were bigger. These amyloids are β-sheet-rich proteins and have already been identified in activated sludge flocs and AGS (7, 9). The production of amyloid has been suggested for ammonia-oxidizing bacteria, *Zoogloea*, and some GAOs, such as *Competibacter*. In this study, the activity of these microorganisms was relatively low, and the transcription of these amyloid adhesin genes could not be detected.

All along the experiment, the fast-settling capacity of the granules remained. Neither the change of the influent composition, the modification of the microbial community, nor the potential change in the EPS composition caused a disintegration of the granules. It could be because of the slow dynamics in these modifications and because *Accumulibacter* remained quite abundant and active. Instead of a disintegration, a modification in the granule shape from smooth to grainy was observed, following the change in the PAO to GAO metabolism and the associated microbial populations. The granule shape can be related to the predominant organisms as described previously (24). However, it could also be a natural maturation of the granules (29), and further investigations correlating image analysis and microbial activity would be of great help to better understand and manage the granule life cycle.

The present metatranscriptomics analysis allowed identification of the microorganisms potentially involved in the production of EPS composing the granule extracellular matrix. Moreover, the analysis indicated that, even when working with an enriched biomass, other populations could be involved in the EPS formation and/or stability. In most of the studies, the role of these flanking communities is often underestimated. Although the primary goal of this study is not to quantify or characterize EPS, future studies would benefit from longitudinal analysis of the EPS in parallel with the EPS producer identification, which would provide a more comprehensive picture of the granule formation. However, technical difficulties, such as biomass washout due to extensive sampling and EPS extraction biases due to the complexity of the EPS, are to be expected (30). Each extraction method has its own pros and cons due to the EPS properties (low-bind, charge…) as reviewed by Huang et al. (31), and combining different methods would improve the precision in determining the composition and limit these biases (31). Additionally, the analyses of the present study were conducted as an overall microbial activity in the reactor during the granulation, but there could be heterogeneity at the granule level. Indeed, as shown by Leventhal et al. (32), individual granules can be composed of a specific microbial community leading to potentially a different EPS composition (32).

### Conclusions

EPS are central in the AGS technology by aggregating a diverse microbial community in a granular biofilm. In this study, *Accumulibacter* was the most active population during granulation and transcribed genes of several EPS pathways. Following a long period with low phosphate concentrations, during which a GAO metabolism was established, *Accumulibacter* was still present and active, but the GAO *Propionivibrio* became predominant. *Propionivibrio* highly transcribed genes involved in the EPS production, but the pattern of transcribed EPS pathways was different from *Accumulibacter*. However, the granules remained intact, indicating that the change in the microbial community composition had no major impact on granule stability. The role of the flanking community, which included the PAO *Azonexus*, the GAO *Contendobacter* and *Competibacter*, *Flavobacterium*, *Rhodoferax*, *Kaistella*, and *Zoogloea*, should not be underestimated, as they contributed to a considerable proportion of the metatranscriptome. Metatranscriptomics analysis represented the ensemble of the reactor biomass at specific stages, but each granule can be composed of different microbial communities and, therefore, contain different EPS. To have a comprehensive view on the granulation process, further analyses should be conducted at granule level and couple EPS measurement to microbial community identification.

## MATERIALS AND METHODS

### Reactor set-up

The experiment was carried out in a bubble column SBR of 6.2 cm diameter and 2.4 L working volume in fill-draw mode. The temperature was regulated at 18°C ± 1°C by recirculating water in the double-wall reactor. The pH was maintained at 7.5 ± 1 by regulating the injection of 1 mM HCl or 1 mM NaOH with an ISFET probe (Endress+Hauser, Switzerland) using proportional–integral–derivative control. The pO2 and conductivity were monitored using two ISFET probes (Endress+Hauser).

A typical cycle corresponded to 5 min of sparging nitrogen gas to ensure anaerobic condition, 60 min feeding under anaerobic mixing condition, 45 min anaerobic phase to ensure total carbon consumption, 150 min aerobic phase by sparging compressed air, 15 min of sparging nitrogen to return to anaerobic condition, 5 min of settling, and 5 min of withdrawal of half of the reactor working volume. The hydraulic retention time was set at 9.5 h, and the solid retention time was set at 21 days by sampling the mixed biomass at the end of the aerobic phase once every week.

The durations of the different phases of the cycle were adapted across the experiment to ensure the optimal functioning of the reactor.

### Inoculum and media

The sludge used as inoculum was collected in the anaerobic tank of ARA Thunersee (Thun, Switzerland), an activated sludge wastewater treatment plant that performs biological phosphorus removal. After transport (2–3 h), the sludge was centrifuged for 10 min at 4°C and 5,000 × *g* to concentrate the biomass. Then, the sludge was homogenized with a glass homogenizer with a distance between the pestle and the tube between 0.15 and 0.25 mm (50 cm^3^, Carl Roth, Germany). The total and volatile solid compositions were measured, and the sludge was stored at 4°C for a maximum of 2 days.

The reactor influent was created by mixing two 8.89 times concentrated solutions of C and NP and Milli-Q water. Concentrated solution C contained 5.67 g/L C_2_H_3_O_2_Na–3H_2_O, 2.28 g/L C_3_H_5_O_2_Na, 0.889 g/L MgSO_4_–7H_2_O, 2.2 g/L MgCl_2_–6H_2_O, and 0.4 g/L CaCl_2_–H_2_O. Concentrated solution NP contained 1.671 g/L K_2_HPO_4_, 0.649 g/L KH_2_PO_4_, and 0.048 g/L C_4_H_8_N_2_S to inhibit the nitrification and 50 mL of trace element solution composed of 16.22 g/L C_10_H_14_N_2_Na_2_O_8_–H_2_O, 0.44 g/L ZnSO_4_–7H_2_O, 1.012 g/L MnCl_2_–4H_2_O, 7.049 g/L (NH_4_)_2_Fe(SO_4_)_2_–6H_2_O, 0.328 g/L (NH_4_)_6_Mo_7_O_24_–4H_2_O, 0.315 g/L CuSO_4_–5H_2_O, and 0.322 g/L CoCl_2_–6H2O. Both solutions were autoclaved in 10 L glass bottles. Before use, 250 mL of bicarbonate solution composed of 0.933 g/L NH_4_HCO_3_ and 0.533 g/L KHCO_3_ was added to the NP solution to reach a final volume of 10 L. At each cycle, 120 mL of concentrated solutions C and NP was mixed with 960 mL of distilled water to feed the reactor and achieve a final chemical oxygen demand (COD) concentration of 300 mgO_2_/L in the SBR.

### Description of the experiment

The reactor was inoculated with the concentrated and homogenized activated sludge to obtain a final concentration of 5 g MLSS/L. To limit the risk of carbon remaining while starting the aerobic phase, the COD concentration was increased stepwise from 50 to 300 mg O_2_/L within 15 days. Moreover, the anaerobic phase was decreased based on COD measurements from 150 min at the beginning of the experiment to 45 min at the end. The settling time was decreased from 50 to 5 min when granule formation and fast biomass settling were observed.

The SBR was operated for 183 days. After 103 days of operation, the COD/P ratio was increased from 12 to 200 by decreasing the phosphate concentration in the influent. Because the phosphate concentration released at the end of the anaerobic phase remained high, two consecutive cycles were operated differently. These conditioning cycles were modified as follows: at the end of the anaerobic phase, the reactor was stopped to allow the biomass to settle, and half of the working volume was removed before the start of the aerobic phase to reduce the formation of polyphosphate reserves. This operation was repeated 1 week later.

### Nutrient removal performance monitoring

Nutrient removal performance of the reactors was measured weekly. Samples of 50 mL were taken from the SBR at the end of the anaerobic and aerobic phases, centrifuged 5 min at room temperature at 4,200 × *g*, and the supernatant was filtered (0.45 µm). A sample of the synthetic reactor influent was also collected and filtered (0.45 µm). The samples were stored at 4°C until further analysis. The concentrations of the anions (P–PO_4_^3−^, N–NO_3_^−^, and N–NO_2_^−^) were measured using ionic chromatography (IC, ICS-90, IonPacAS14A column) with an electrical conductivity detector (Dionex, Switzerland). The COD was measured by spectrophotometry using two different kits: LCK514 (100–2,000 mgO_2_/L) and LCK 314 (15–150 mgO_2_/L) (Hach, USA) measured on a spectrophotometer DR 3900 (Hach, USA).

The total and volatile solids were determined in the sludge obtained by centrifuging 100 mL of mixed liquor reactor sample taken at the end of the aerobic phase. The mass of the dried pellet after 12 h of drying at 105°C yielded the total solids, and the mass loss after 2 h of calcination at 550°C resulted in volatile solids.

### AGS granule size and morphology

Granule formation was followed by capturing a picture of the biomass using a camera from a Dino-Lite Edge Digital Microscope and DinoCapture 2.0 software (AnMo Electronics Corporation, Taiwan). The mean particle size distribution was measured weekly using an LS13 320 Series particle size analyzer (Beckman Coulter, Germany) connected to a universal liquid module. The particle range goes from 0.37 to 2,000 µm, and the following parameters were used for the measurements: obscuration 12%, optical model Fraunhofer, run length of 60 s, and pump flow of 20%.

### 16S rRNA gene amplicon sequencing and analysis

The sampling for the 16S rRNA gene amplicon sequencing, the DNA extraction, and 16S rRNA gene amplicon sequencing (protocol no. 2) were performed as described by Adler and Holliger (33). Briefly, the samples were taken every week at the end of the anaerobic phase, washed with phosphate-buffered saline 1× solution, homogenized with a glass pestle, and stored at −20°C until use. The extraction was performed using the automated robot 16 DNA purification system (Maxwell, Promega Corporation, Switzerland) after enzymatic lysis (lysozyme, 1 h at 37°C). The bacterial 16S rRNA gene hypervariable regions V1–V2 were amplified using the universal primers 27F and 338R and high-fidelity Q5 polymerase (high-fidelity 2× Master Mix, Biolabs, Inc., USA). A secondary indexing PCR was performed on normalized samples at 5 ng/µL using TG Nextera XT Index Kit v2 Set B (#FC-131-2002, Illumina, USA). The products were purified using Agencourt AMPure XP magnetic beads and quantified using Qubit dsDNA HS. Pooling of the normalized samples was performed at 10 nM, and sequencing was performed by the Lausanne Genomic Technologies Facility (University of Lausanne, Switzerland) on an Illumina MiSeq v2 in paired-end mode (2 × 250).

Adapter sequences were removed from the reads using cutadapt v3.5 (34) and default parameters after removing Ns from the sequences using DADA2 v1.32.0 (35) with the filterAndTrim function. Then, reads were quality-filtered and trimmed using the filterAndTrim function (truncLen = c(225, 225), maxEE = c(2, 2), truncQ = 2, maxN = 0). Error rates were estimated for both forward and reverse sequences using learnErrors (nbases = 1e10, randomize = TRUE). Sequences were dereplicated and inferred using the derepFastq and dada (pool = "pseudo") functions. Paired-end sequences were merged using the mergePairs function before removing the chimera with removeBimeraDenovo (method = "consensus"). Finally, the taxonomy was assigned using the assignTaxonomy function with the MIDAS 5.3 database (36). In total, 7,138 ASVs were obtained.

The obtained ASVs were agglomerated to the genus level using the glom_tax function of the phyloseq package (1.46.0). After agglomeration, 1,002 genera remained. To visualize the most abundant microorganisms, the genera with a minimum of 3% in at least one sample were kept (28 genera). The full taxonomic information and counts of the genera are provided in Appendix B.

### Metatranscriptomics

For the metatranscriptomics analysis, four biomass samples were collected on days 13, 26, 103, and 182 to capture the microbial activity at different stages of granule formation. An aliquot of 15 mL of mixed liquor was sampled after 15 min of feeding and after 15 min of aeration on three consecutive cycles to get biological replicates. The samples were quickly placed on ice and centrifuged for 1 min at 4°C and 4,200 × *g*. The supernatant was filtered (0.45 µm) to measure the COD and P. The pellet was resuspended in two volumes (g pellet:mL volume) of RNA protect tissue (Qiagen, Germany) and homogenized by passing three times through a needle (26G). After an incubation at room temperature (RT) for 5 min, the sample was centrifuged for 5 min at RT and 5,000 × *g*, and the supernatant was discarded. The pellet was snap-frozen in liquid nitrogen and stored at −80°C until RNA extraction.

For RNA extraction, the samples were thawed on ice, resuspended in 0.5 mL of TRIzol (#15596-0026, Invitrogen, Fisher Scientific AG, Switzerland), and incubated for 5 min at RT. Then, 0.1 mL of chloroform 99+% was added, and the mixture was vortexed for 15 s and incubated 2 min at RT before being centrifuged 15 min at 15,500 × *g* at 4°C. The upper portion was recovered and mixed with 400 µL of 100% ethanol. RNA was purified using an RNA purification kit (Direct-zol RNA Miniprep #R2050, Zymo Research, Germany) following the manufacturer’s recommendations, with the exception of centrifugation performed for 1 min at 13,000 × *g*. DNA was removed using a TURBO DNA-*free* Kit (#AM1907, Thermo Fisher Scientific, Switzerland) following the manufacturer’s recommendations. RNA was purified using magnetic beads (Agencourt RNACleaner XP, #A63987, Beckman Coulter) by adding 76 µL magnetic beads to the extracted RNA. After mixing 10 times up and down, the samples were incubated at RT for 5 min. The samples were placed on the magnetic rack for 10 min before removing the supernatant, followed by the addition of 200 µL of ethanol 70% and incubation of 30 s. The ethanol was removed, and the previous steps were repeated twice. After removing the ethanol, 32 µL of RNase and DNase-free water was added to the pellet out of the rack and resuspended 10 times by up and down. The samples were incubated for 1 min before being returned to the rack for 1 min. Finally, the supernatants were collected. Bacterial ribosomal RNA (rRNA) was removed using a QiaSeq FastSelect 5S/16S/23S Kit (#335925, Qiagen) following the manufacturer’s recommendations on 1 µg of total RNA (protocol with TruSeq stranded library preparation) with the following modification: the first step of combined fragmentation and hybridization was performed for 1 min at 89°C. Libraries were then generated using the TruSeq Stranded mRNA Sample Preparation Kit (#20020594, Illumina, USA) and IDT for Illumina TruSeq RNA UD Indexes (#20022371, Illumina) following the reference guide #1000000040498 for the LS procedure without optional steps. For the clean-up amplified DNA step, the ratio of magnetic beads:PCR products was 0.7, and 20 µL of RSB was added to release the genetic material from the bead. The amplification was quantified with Qubit dsDNA HS Assay Kit (#Q32854, Life Technologies), and the quality was checked by electrophoresis using Agilent High Sensitivity DNA Kit (#5067-4626, Agilent Technologies). The concentrations of the samples were normalized to 10 nM and pooled. Sequencing analysis was performed at the Lausanne Genomic Technologies Facility, University of Lausanne (Switzerland) on a NovaSeq 6000 in paired-end mode (2 × 150).

Before *de novo* metatranscriptomics analysis, the quality of the reads was evaluated using FastQC v0.11.9 (https://www.bioinformatics.babraham.ac.uk/projects/fastqc/) for the raw and at each step of the treatment. The results were summarized using MultiQC v1.13 (37). The reads were filtered and trimmed using BBDuk from BBMap v39.01 (https://jgi.doe.gov/data-and-tools/software-tools/bbtools/bb-tools-user-guide/bbmap-guide/) using the following parameters: *ktrim* = *r*, *k =* 23, *mink* = 11, *hdist* = 1, *tpe*, *tbo* for the adapter trimming steps and *qtrim* = *rl*, *trimq* = 20, *minlen* = 50, *maq* = 20, *maxns* = 1 for the quality trimming and filtering. Ribosomal RNA was removed using sortMeRNA v4.3.6 (38) and the databases for silva-bac-16s-id90, silva-arc-16s-id95, silva-euk-18s-id95, silva-bac-23s-id98, silva-arc-23s-id98, silva-euk-28s-id98, rfam-5s-database-id98, rfam-5.8s-database-id98 from Silva and rfam. RNASpades (39) was used to co-assemble the reads, and the results from the hard filtering from RNASpades were used for the following steps. The quality of the obtained assembly was evaluated using BUSCO 5.4.7, bacteria_Odb10.2020–03-06 (*-m transcriptome*) (40) (complete: 65.3% [single-copy: 18.5%, duplicated: 46.8%], fragmented: 7.3%, missing: 27.4%, n:124), and bowtie2 v2.4.1 (41) (*-k 20*) by mapping back the reads onto the co-assembly (95% of reads mapped onto the assembly). The redundancy in the sequences was considered by clustering the redundant information using CD-HIT v4.8.1 (42) (*-c 0.95, -n 10, -d 0, -M 16000*). FeatureCount v2.0.1 (43) was used to summarize the count in a table using the following parameters *-O -M -C -B –fraction* (360,881 annotated genes). The pipeline results are summarized in Appendix C. DRAM v1.4.6 (44) and eggNOG-mapper v2.1.11 (45) were used for gene prediction and annotation. For the transcripts of interest highlighted in this paper, the amino acid sequences were blasted on National Center for Biotechnology Information using BLASTP to check the taxonomic assignment and gene description. If the percentage of identity was under 80%, the transcript was not considered for further analysis. Kaiju v1.9.9 and the non-redundant protein database (update 22 March 2022) (46) were used to obtain taxonomic assignments at the genus level of the assemblies. Gene and taxonomic information are summarized in Appendix D. mOTUs v3 (47) was used to obtain the expression profile of the microbial community at the species level. Publicly available (48–50) and in-house (51) metagenome-assembled genomes were added to the mOTUs database v2.8 following the procedure described in the GitHub page (https://github.com/motu-tool/mOTUs-extender). This step was done in order to extend the mOTUs database with genomes from similar biosystems than our study and increase genomes from wastewater, EBPR, and AGS systems.

### Microbial community activity analysis

All the statistical analyses were done on R (v4.3.3) and RStudio (2023.12.1+402), and the graphics were obtained using ggplot2 (v3.5.1). Low count filtering (minimum: 15 counts) was applied using *filterByExpr* from edgeR (v4.0.16) (52) on the metatranscriptomics count results. After filtering, 184,899 genes remained. The expression of each transcript was normalized using a trimmed mean of *M*-values with the *normLibSizes* (method = “TMM”) function from edgeR. The results of the raw, filtered, and normalized counts are provided in Zenodo at https://zenodo.org/records/14710676.

The normalized count values were transformed into count per million (cpm) using the *cpm* function from edgeR. The relative activity of the microbial community was obtained by summing the cpm values to the genus level for each sample. Moreover, the Shannon index to compare the diversity between the time points was calculated at genus level on this data set.

Genes involved in specific metabolic pathways were selected based on KO, COG, and PFAM identification numbers. The complete list of the genes of interest is provided in Appendix E. For these selected transcripts, the non-normalized and non-transformed count values were summed to summarize the result to KO and genus level for each sample. The data were subsequently TMM-normalized and used for the differential gene expression analysis performed using edgeR. A gene was considered as differentially transcribed if the adjusted *P* value (function *topTags* with Bonferroni correction for multiple comparisons) was lower than 0.01, and if the absolute log2FC was higher than 2.

The analysis was done to compare the gene transcription between the time points. However, the phases were kept separate for the comparison of each time point, and the cycles were considered as replicates for each sample. Only the results for the feeding samples are presented in the main article, as both results for feeding and aerobic conditions were highly similar for the analyzed genes. The results for the aerobic samples are presented in Appendix A, Figures A2 and A3.

## Data Availability

Raw metatranscriptomics reads and the *de novo* assembly after clustering using CD-HIT have been deposited at the Sequence Read Archive (SRA) and Transcriptome Shotgun Assembly (TSA) under the BioProject ID PRJNA1144857. The raw reads from the 16S rRNA gene amplicon sequencing are available under the BioProject ID PRJNA1125294. The supplemental materials are available in Zenodo server under the link https://zenodo.org/records/14710676. The scripts for the DADA2 pipeline, for the bioinformatic pipeline, and to make the analyses and the figures are available in the Zenodo server as well.

## SUPPLEMENTAL MATERIAL

Appendix A aem.01769-24-s0001.docx Biomass pictures and gene transcription in the aerobic phase.

Appendix B aem.01769-24-s0002.xlsx Summary of the 16S rRNA gene amplicon sequencing.

Appendix C aem.01769-24-s0003.xlsx Summary of the bioinformatic results.

Appendix D aem.01769-24-s0004.csv Gene annotation.

Appendix E aem.01769-24-s0005.csv List of genes of interest.

ASM does not own the copyrights to Supplemental Material that may be linked to, or accessed through, an article. The authors have granted ASM a non-exclusive, world-wide license to publish the Supplemental Material files. Please contact the corresponding author directly for reuse.

## References

[1] Nielsen PH, McIlroy SJ, Albertsen M, Nierychlo M. 2019. Re-evaluating the microbiology of the enhanced biological phosphorus removal process. Curr Opin Biotechnol 57:111–118. doi:10.1016/j.copbio.2019.03.008 30959426

[2] Rosa-Masegosa A, Muñoz-Palazon B, Gonzalez-Martinez A, Fenice M, Gorrasi S, Gonzalez-Lopez J. 2021. New advances in aerobic granular sludge technology using continuous flow reactors: engineering and microbiological aspects. Water (Basel) 13:1792. doi:10.3390/w13131792

[3] Flemming H-C, van Hullebusch ED, Neu TR, Nielsen PH, Seviour T, Stoodley P, Wingender J, Wuertz S. 2023. The biofilm matrix: multitasking in a shared space. Nat Rev Microbiol 21:70–86. doi:10.1038/s41579-022-00791-0 36127518

[4] Nouha K, Yan S, Tyagi RD, Surampalli RY. 2016. EPS producing microorganisms from municipal wastewater activated sludge. J Pet Environ Biotechnol 07. doi:10.4172/2157-7463.1000255

[5] Dueholm MKD, Besteman M, Zeuner EJ, Riisgaard-Jensen M, Nielsen ME, Vestergaard SZ, Heidelbach S, Bekker NS, Nielsen PH. 2023. Genetic potential for exopolysaccharide synthesis in activated sludge bacteria uncovered by genome-resolved metagenomics. Water Res 229:119485. doi:10.1016/j.watres.2022.119485 36538841

[6] Seviour TW, Lambert LK, Pijuan M, Yuan Z. 2011. Selectively inducing the synthesis of a key structural exopolysaccharide in aerobic granules by enriching for Candidatus “Competibacter phosphatis”. Appl Microbiol Biotechnol 92:1297–1305. doi:10.1007/s00253-011-3385-1 21670976

[7] Lin Y, Reino C, Carrera J, Pérez J, van Loosdrecht MCM. 2018. Glycosylated amyloid-like proteins in the structural extracellular polymers of aerobic granular sludge enriched with ammonium-oxidizing bacteria. Microbiologyopen 7:e00616. doi:10.1002/mbo3.616 29604180 PMC6291783

[8] Tomás-Martínez S, Zwolsman EJ, Merlier F, Pabst M, Lin Y, van Loosdrecht MCM, Weissbrodt DG. 2022. Turnover of the extracellular polymeric matrix in an EBPR microbial community. Microbiology. doi:10.1101/2022.08.11.503576 PMC10006046 36759376

[9] Christiaens A-S, Van Steenkiste M, Rummens K, Smets I. 2022. Amyloid adhesin production in activated sludge is enhanced in lab-scale sequencing batch reactors: Feeding regime impacts microbial community and amyloid distribution. Water Res X 17:100162. doi:10.1016/j.wroa.2022.100162 36479239 PMC9720597

[10] Singleton CM, Petriglieri F, Wasmund K, Nierychlo M, Kondrotaite Z, Petersen JF, Peces M, Dueholm MS, Wagner M, Nielsen PH. 2022. The novel genus, “Candidatus Phosphoribacter”, previously identified as Tetrasphaera, is the dominant polyphosphate accumulating lineage in EBPR wastewater treatment plants worldwide. ISME J 16:1605–1616. doi:10.1038/s41396-022-01212-z 35217776 PMC9123174

[11] Ford RC, Brunkan-LaMontagne AL, Collins RF, Clarke BR, Harris R, Naismith JH, Whitfield C. 2009. Structure-function relationships of the outer membrane translocon Wza investigated by cryo-electron microscopy and mutagenesis. J Struct Biol 166:172–182. doi:10.1016/j.jsb.2009.02.005 19236919 PMC3498625

[12] Weissbrodt DG. 2024. Factors Selecting for Polyphosphate- and Glycogen-Accumulating Organisms in Granular Sludge Sequencing Batch Reactors, p 397–424. In Engineering granular microbiomes. Springer, Cham. 10.1016/j.watres.2013.08.043 24200006

[13] Tan CH, Koh KS, Xie C, Tay M, Zhou Y, Williams R, Ng WJ, Rice SA, Kjelleberg S. 2014. The role of quorum sensing signalling in EPS production and the assembly of a sludge community into aerobic granules. ISME J 8:1186–1197. doi:10.1038/ismej.2013.240 24430488 PMC4030234

[14] Flowers JJ, Cadkin TA, McMahon KD. 2013. Seasonal bacterial community dynamics in a full-scale enhanced biological phosphorus removal plant. Water Res 47:7019–7031. doi:10.1016/j.watres.2013.07.054 24200007 PMC4520395

[15] Adler A, Poirier S, Pagni M, Maillard J, Holliger C. 2022. Disentangle genus microdiversity within a complex microbial community by using a multi-distance long-read binning method: example of Candidatus Accumulibacter. Environ Microbiol 24:2136–2156. doi:10.1111/1462-2920.15947 35315560 PMC9311429

[16] Petriglieri F, Singleton C, Peces M, Petersen JF, Nierychlo M, Nielsen PH. 2021. “Candidatus Dechloromonas phosphoritropha” and “Ca. D. phosphorivorans”, novel polyphosphate accumulating organisms abundant in wastewater treatment systems. ISME J 15:3605–3614. doi:10.1038/s41396-021-01029-2 34155336 PMC8630035

[17] Choi A, Cha IT, Lee KE, Son YK, Yu J, Seol D. 2023. The role of Flavobacterium enshiense R6S-5-6 in the wetland ecosystem revealed by whole-genome analysis. Curr Microbiol 80:83. doi:10.1007/s00284-022-03157-0 36680647 PMC9867689

[18] Liu Q, Liu H-C, Zhou Y-G, Xin Y-H. 2019. Microevolution and adaptive strategy of psychrophilic species Flavobacterium bomense sp. nov. Isolated From Glaciers. Front Microbiol 10:449872. doi:10.3389/fmicb.2019.01069 PMC6538692 31178833

[19] Dueholm MKD, Nierychlo M, Andersen KS, Rudkjøbing V, Knutsson S, MiDAS Global Consortium, Albertsen M, Nielsen PH. 2022. MiDAS 4: a global catalogue of full-length 16S rRNA gene sequences and taxonomy for studies of bacterial communities in wastewater treatment plants. Nat Commun 13:1908. doi:10.1038/s41467-022-29438-7 35393411 PMC8989995

[20] Maszenan AM, Bessarab I, Williams RBH, Petrovski S, Seviour RJ. 2022. The phylogeny, ecology and ecophysiology of the glycogen accumulating organism (GAO) Defluviicoccus in wastewater treatment plants. Water Res 221:118729. doi:10.1016/j.watres.2022.118729 35714465

[21] Petriglieri F, Singleton CM, Kondrotaite Z, Dueholm MKD, McDaniel EA, McMahon KD, Nielsen PH. 2022. Reevaluation of the phylogenetic diversity and global distribution of the genus “Candidatus Accumulibacter”. mSystems 7:e0001622. doi:10.1128/msystems.00016-22 35467400 PMC9238405

[22] de Kreuk MK, van Loosdrecht MCM. 2004. Selection of slow growing organisms as a means for improving aerobic granular sludge stability. Water Sci Technol 49:9–17. doi: 10.2166/wst.2004.0792 15303717

[23] Acevedo B, Oehmen A, Carvalho G, Seco A, Borrás L, Barat R. 2012. Metabolic shift of polyphosphate-accumulating organisms with different levels of polyphosphate storage. Water Res 46:1889–1900. doi:10.1016/j.watres.2012.01.003 22297158

[24] Weissbrodt DG, Neu TR, Kuhlicke U, Rappaz Y, Holliger C. 2013. Assessment of bacterial and structural dynamics in aerobic granular biofilms. Front Microbiol 4:175. doi:10.3389/fmicb.2013.00175 23847600 PMC3707108

[25] Livingstone PG, Morphew RM, Cookson AR, Whitworth DE. 2018. Genome analysis, metabolic potential, and predatory capabilities of Herpetosiphon llansteffanense sp. nov. Appl Environ Microbiol 84. doi:10.1128/AEM.01040-18 PMC6210107 30194103

[26] de Graaff DR, Felz S, Neu TR, Pronk M, van Loosdrecht MCM, Lin Y. 2019. Sialic acids in the extracellular polymeric substances of seawater-adapted aerobic granular sludge. Water Res 155:343–351. doi:10.1016/j.watres.2019.02.040 30852321

[27] Tomás-Martínez S, Kleikamp HBC, Neu TR, Pabst M, Weissbrodt DG, van Loosdrecht MCM, Lin Y. 2021. Production of nonulosonic acids in the extracellular polymeric substances of “Candidatus Accumulibacter phosphatis”. Appl Microbiol Biotechnol 105:3327–3338. doi:10.1007/s00253-021-11249-3 33791836 PMC8053191

[28] Zahra SA, Abdullah N, Iwamoto K, Yuzir A, Mohamad SE. 2022. Alginate-like exopolysaccharides in aerobic granular sludge: a review. Mater Today 65:3046–3053. doi:10.1016/j.matpr.2022.04.032

[29] Mills S, Trego AC, Prevedello M, De Vrieze J, O’Flaherty V, Lens PNL, Collins G. 2024. Unifying concepts in methanogenic, aerobic, and anammox sludge granulation. Environ Sci Ecotechnol 17:100310. doi:10.1016/j.ese.2023.100310 37705860 PMC10495608

[30] Cydzik-Kwiatkowska A. 2021. Biopolymers in aerobic granular sludge—their role in wastewater treatment and possibilities of re-use in line with circular economy. Energies 14:7219. doi:10.3390/en14217219

[31] Huang L, Jin Y, Zhou D, Liu L, Huang S, Zhao Y, Chen Y. 2022. A review of the role of extracellular polymeric substances (EPS) in wastewater treatment systems. IJERPH 19:12191. doi:10.3390/ijerph191912191 36231490 PMC9566195

[32] Leventhal GE, Boix C, Kuechler U, Enke TN, Sliwerska E, Holliger C, Cordero OX. 2018. Strain-level diversity drives alternative community types in millimetre-scale granular biofilms. Nat Microbiol 3:1295–1303. doi:10.1038/s41564-018-0242-3 30250246

[33] Adler A, Holliger C. 2020. Multistability and reversibility of aerobic granular sludge microbial communities upon changes from simple to complex synthetic wastewater and back. Front Microbiol 11:574361. doi:10.3389/fmicb.2020.574361 33324361 PMC7726351

[34] Martin M. 2011. Cutadapt removes adapter sequences from high-throughput sequencing reads. EMBnet j 17:10. doi:10.14806/ej.17.1.200

[35] Callahan BJ, McMurdie PJ, Rosen MJ, Han AW, Johnson AJA, Holmes SP. 2016. DADA2: High-resolution sample inference from Illumina amplicon data. Nat Methods 13:581–583. doi:10.1038/nmeth.3869 27214047 PMC4927377

[36] Dueholm MKD, Andersen KS, Petersen A-KC, Rudkjøbing V, Alves M, Bajón-Fernández Y, Batstone D, Butler C, Cruz MC, Davidsson Å, et al.. 2024. MiDAS 5: global diversity of bacteria and archaea in anaerobic digesters. bioRxiv. doi:10.1101/2023.08.24.554448 PMC11199495 38918384

[37] Ewels P, Magnusson M, Lundin S, Käller M. 2016. MultiQC: summarize analysis results for multiple tools and samples in a single report. Bioinformatics 32:3047–3048. doi:10.1093/bioinformatics/btw354 27312411 PMC5039924

[38] Kopylova E, Noé L, Touzet H. 2012. SortMeRNA: fast and accurate filtering of ribosomal RNAs in metatranscriptomic data. Bioinformatics 28:3211–3217. doi:10.1093/bioinformatics/bts611 23071270

[39] Bushmanova E, Antipov D, Lapidus A, Prjibelski AD. 2019. rnaSPAdes: a de novo transcriptome assembler and its application to RNA-Seq data. Gigascience 8:1–13. doi:10.1093/gigascience/giz100 PMC6736328 31494669

[40] Manni M, Berkeley MR, Seppey M, Zdobnov EM. 2021. BUSCO: assessing genomic data quality and beyond. Curr Protoc 1:e323. doi:10.1002/cpz1.323 34936221

[41] Langmead B, Salzberg SL. 2012. Fast gapped-read alignment with Bowtie 2. Nat Methods 9:357–359. doi:10.1038/nmeth.1923 22388286 PMC3322381

[42] Li W, Godzik A. 2006. Cd-hit: a fast program for clustering and comparing large sets of protein or nucleotide sequences. Bioinformatics 22:1658–1659. doi:10.1093/bioinformatics/btl158 16731699

[43] Liao Y, Smyth GK, Shi W. 2014. featureCounts: an efficient general purpose program for assigning sequence reads to genomic features. Bioinformatics 30:923–930. doi:10.1093/bioinformatics/btt656 24227677

[44] Shaffer M, Borton MA, McGivern BB, Zayed AA, La Rosa SL, Solden LM, Liu P, Narrowe AB, Rodríguez-Ramos J, Bolduc B, Gazitúa MC, Daly RA, Smith GJ, Vik DR, Pope PB, Sullivan MB, Roux S, Wrighton KC. 2020. DRAM for distilling microbial metabolism to automate the curation of microbiome function. Nucleic Acids Res 48:8883–8900. doi:10.1093/nar/gkaa621 32766782 PMC7498326

[45] Cantalapiedra CP, Hernández-Plaza A, Letunic I, Bork P, Huerta-Cepas J. 2021. eggNOG-mapper v2: functional annotation, orthology assignments, and domain prediction at the metagenomic scale. Mol Biol Evol 38:5825–5829. doi:10.1093/molbev/msab293 34597405 PMC8662613

[46] Menzel P, Ng KL, Krogh A. 2016. Fast and sensitive taxonomic classification for metagenomics with Kaiju. Nat Commun 7:11257. doi:10.1038/ncomms11257 27071849 PMC4833860

[47] Ruscheweyh H-J, Milanese A, Paoli L, Karcher N, Clayssen Q, Keller MI, Wirbel J, Bork P, Mende DR, Zeller G, Sunagawa S. 2022. Cultivation-independent genomes greatly expand taxonomic-profiling capabilities of mOTUs across various environments. Microbiome 10:212. doi:10.1186/s40168-022-01410-z 36464731 PMC9721005

[48] Ye L, Mei R, Liu W-T, Ren H, Zhang X-X. 2020. Machine learning-aided analyses of thousands of draft genomes reveal specific features of activated sludge processes. Microbiome 8:16. doi:10.1186/s40168-020-0794-3 32046778 PMC7014675

[49] Singleton CM, Petriglieri F, Kristensen JM, Kirkegaard RH, Michaelsen TY, Andersen MH, Kondrotaite Z, Karst SM, Dueholm MS, Nielsen PH, Albertsen M. 2021. Connecting structure to function with the recovery of over 1000 high-quality metagenome-assembled genomes from activated sludge using long-read sequencing. Nat Commun 12:2009. doi:10.1038/s41467-021-22203-2 33790294 PMC8012365

[50] Arumugam K, Bessarab I, Haryono MAS, Liu X, Zuniga-Montanez RE, Roy S, Qiu G, Drautz-Moses DI, Law YY, Wuertz S, Lauro FM, Huson DH, Williams RBH. 2021. Recovery of complete genomes and non-chromosomal replicons from activated sludge enrichment microbial communities with long read metagenome sequencing. NPJ Biofilms Microbiomes 7:23. doi:10.1038/s41522-021-00196-6 33727564 PMC7966762

[51] Saini JS, Adler A, Cardona L, Rodilla Ramírez PN, Pei R, Holliger C. 2024. Microbial genome collection of aerobic granular sludge cultivated in sequencing batch reactors using different carbon source mixtures. Microbiol Resour Announc 13:e0010224. doi:10.1128/mra.00102-24 38534152 PMC11080561

[52] Robinson MD, McCarthy DJ, Smyth GK. 2010. edgeR: a bioconductor package for differential expression analysis of digital gene expression data. Bioinformatics 26:139–140. doi:10.1093/bioinformatics/btp616 19910308 PMC2796818

