# Supplementary material for: Multiple extracellular polymeric substance pathways transcribed by Accumulibacter and the flanking community during aerobic granule formation and after influent modification
Source: Appl Environ Microbiol. 2025 Mar 31;91(4):e01769-24. doi: 10.1128/aem.01769-24 (PMC12016536; doi:10.1128/aem.01769-24)
Supplement: Appendix A — Biomass pictures and gene transcription in the aerobic phase. [file aem.01769-24-s0001.docx]

**Supplementary material**

**Multiple extracellular polymeric substances pathways expressed by Accumulibacter and the flanking community during aerobic granule formation and after influent modification**

^1^*Laëtitia Cardona, ^1^Jaspreet Singh Saini, ^1^Pilar Natalia Rodilla Ramírez, ^1^Aline Adler, ^1^Christof Holliger

^1^Laboratory for Environmental Biotechnology, Ecole Polytechnique Fédérale de Lausanne, Lausanne, Switzerland

*corresponding author: Laetitia.cardona@epfl.ch

Address

EPFL - Ecole Polytechnique Federale de Lausanne

ENAC IIE LBE

CH B2 407

Station 6

1015 Lausanne - Switzerland


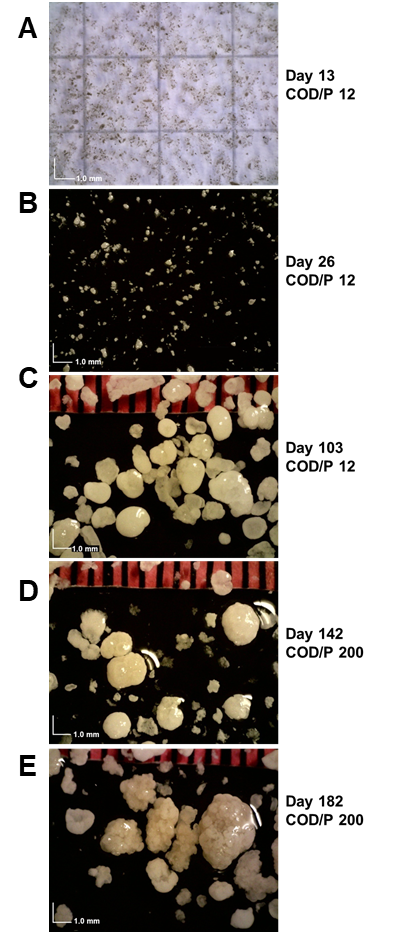


Supplementary figure A1. Evolution of the biomass before and after decreasing the phosphate concentration in influent composition to reach a COD/P ratio of 200. A) After 13 days of operation (COD/P 12). B) After 26 days of operation (COD/P 12). C) After 103 days of operation (COD/P 12). D) After 142 days of operation (COD/P 200). E) After 182 days of operation (COD/P 200)

**Supplementary Figure A2. Transcription of Enhanced Biological Phosphate Removal related genes at the aerobic phase.** Level of expression (log(cpm)) of genes per day for different genera. Differential gene expression analysis was done between two time points (26 versus 13, 103 versus 26 and 182 versus 103) and the significant differences (log-fold change > 2 and pvalue < 0.01) are represented by a triangle (up-pointing for **up regulation** and down-pointing triangle for **down-regulation**).

Supplementary Figure A3. Transcription of biofilm related genes at the aerobic phase. Level of expression (log(cpm)) of genes per day for different genera. Differential gene expression analysis was done between two time points (26 versus 13, 103 versus 26 and 182 versus 103) and the significant differences (log-fold change > 2 and pvalue < 0.01) are represented by a triangle (up-pointing for up regulation and down-pointing triangle for down-regulation).
